# Memoirs of the Royal Medical Society at Paris, Vol. III

**Published:** 1784

**Authors:** 


II. *Memoirs of the Royal Medical Society at Paris, vol.* III. *(continued from page* 253.)

*AN account of the means of obviating, by remedies equally ſimple, eaſy, and efficacious, certain bad effects of the ſmall-pox and meaſles, which frequently occur when thoſe diſorders are of a malignant kind. By* M. de Laſſone.—We have here an account of the efficacy of milk in the diarrhœa which accompanies the ſmall-pox and meaſles; and in the former of theſe diſeaſes, diseases, we are advised to bathe the eyes with rose water, to prevent the eruption of pustules on those parts.—9. *An essay concerning the fat of the human body, and its effects; together with an account of the depravities it is liable to, and the diseases these may give rise to. By* M. Lorry.— This elaborate essay, which extends through upwards of sixty pages, is divided into three parts. In the first, the author treats of the fat in its natural state; in the second, he describes the diseases which a vitiated state of this substance may occasion; and in the third, he considers the particular depravities to which it is liable in different parts of the body. M. Lorry shews that the fat consists of oily and mucilaginous parts, and that, in young animals, the latter is in the greatest proportion. Young persons, he remarks, lose and regain their fat sooner than old ones; and in the latter the fat is yellowish, very oily, in smaller quantity, and of little consistence.—Our author takes notice of the affinity between the fat and the bile; he shews, that the bile of certain birds is purely resinous, and that, in these animals, a little of any acid mixed with the fat, converts it into a resin. He mentions instances of persons, whose skin acquires a yellowish hue after their meals; and observes, that the flesh of full-fed poultry is yellow and bilious.—10. *An account of the experiments made by M. M. de Jussieu, de Lalouette, Jeanroy, and Hallé, the committee appointed to ascertain the properties and effects of the root of the* Plumbago Europæa *in the cure of the itch. By* M. Hallé.—The society, in 1778, having offered a premium for the best method of curing the itch, it was adjudged in favour of M, Sumeire, Physician at Marignan, in Provence. The remedy he proposed is a particular preparation of the root of the *Plumbago Europæa* Linn. All the botanic writers who have described this plant, speak of it as having an acrid caustic quality, It has long been employed in Provence against the itch. Garidel mentions it in his history of the plants that grow about Aix; but what he says of its acrimony tends rather to dissuade from than to recommend its use, as he had seen it bring on a general inflammation of the skin and violent fever. A similar observation was made by Sauvages in the *Mem. de l'academie des sciences* for 1739.

M. Sumeire's manner of preparing this remedy consists in pounding two or three good handfuls of the root, and a small handful of common salt, in a marble mortar, and then pouring on these these ingredients at least a pound of boiling oil of olives. The whole of this mixture, after it has been well stirred for three or four minutes, is to be put on a piece of linen, and when the oil has passed through the linen, the root is to be pressed, and only part of it left in the cloth, which is to be tied in the form of a knot; with this knot, after it has been well soaked in the hot oil, we are directed to rub the whole surface of the body. This friction is to be repeated every twelve hours, till the disease disappears, and we are particularly cautioned to make the oil very hot each time.—The first friction, we are told, generally increases the eruption, but the third or fourth commonly removes the complaint. This mode of treatment, it seems, was first employed by a quack, about forty years ago, and is said to be equally efficacious in cases of tinea. The committee, after trying its effects in several cases, allow that it answers the character given of it, and that it cures without any internal preparation, and more speedily than any other known remedy. M. Vicary, Physician at Avignon, has since informed the society, that the root of the *clematis vitalba*, employed in the same manner, possesses similar virtues.—

11. *On a new manner of preparing the acid ſoaps, and on their uſe in Phyſic. By M.* Cornette.—Acid ſoaps are made by combining any of the mineral acids with oils; but the vitriolic is ſaid to mix with them more eaſily than any other.—The proceſs recommended by our author for preparing a ſoap of this kind, conſiſts in putting four ounces of olive oil into a glaſs or marble mortar, and pouring on it gradually, and at different times, two ounces and a half of concentrated vitriolic acid, taking care to keep the mixture conſtantly ſtirred, till it has acquired a conſiderable degree of conſiſtence. M. Cornette obſerves, that if the proceſs is cautiouſly and ſlowly conducted, no heat is generated, nor any ſulphureous vapour raiſed; a proof, he thinks, that the oil is not changed by the operation. The ſuperabundant acid may be removed, we are told, by two means, either by expoſing the maſs to a damp air, the moiſture of which will be attracted by the acid, or by pouring on it boiling diſtilled water, as M. Achard adviſes. When the latter method is adopted, the ſoap liquefies, and floats on the ſurface of the water, which is to be decanted when cold, and the ſame proceſs is to be then repeated, if there ſhould be ſtill a ſuperabundance dance of acid. M. Cornette informs us, that ſoap, thus prepared, becomes white after a certain time, and that it renders water milky, like common ſoap, and diſſolves in ſpirit of wine. When eſſential oils are employed for this purpoſe, the proceſs, we are told, is more difficult, on account of the heat that is generated; but our author has contrived to obviate this difficulty, by mixing the ingredients in a bath of ſea ſalt and powdered ice. Soaps, thus prepared with eſſential oils, are ſaid to preſerve the ſmell peculiar to the oil that is employed, and are of a brown colour. When they have been recently prepared, they are ſoluble in water; but become leſs ſo, and in ſome degree reſinous, after a certain time. With regard to the medicinal properties of acid ſoaps, M. Cornette informs us, that he has given, with ſucceſs, the olive oil ſoap, in a caſe of nephritic cholic, in doſes of four grains twice a day; in this inſtance, it brought on a copious diſcharge of urine. He aſſures us, that ſome urinary calculi, after reſiſting the alkaline, have yielded to a courſe of the acid ſoaps. The author alſo mentions the good effects of this remedy, in two caſes of ſchirrous tumours.——12. *On the hydrocephalus internus, or dropſy of*

*the ventricles of the brain. By M.* Odier, *Physician at Geneva.* —— From this memoir we learn, that, on an average, twelve or thirteen children die annually of this disease at Geneva. The description our author gives of it is chiefly from Whytt; and in the cure, he lays great stress on the use of blisters. He makes no mention of the use of mercury, administered so as to excite a salivation. He has seen some instances of recovery, which are related; but these, and perhaps we may venture to say, every other case of the kind hitherto published, are of a doubtful nature. M. Odier mentions twelve other cases that have occurred to him, in which the disease proved fatal; and in such of these as were submitted to dissection after death, water was found in the ventricles of the brain. One of these patients was a man, 35 years old, whose symptoms were such as usually accompany hydrocephalus. In this case, on dissection, a large loose hydatid, with a round hole in it, was found in the right ventricle.—Among the remote causes of hydrocephalus, our author enumerates eruptive diseases, such as small-pox, measles, and scarlatina. He has four times seen it come on soon after diseases of this kind. In some cases, he has been inclined to think, that that the abuſe of vomits might have contributed to bring on the diſeaſe; but in each of theſe inſtances there appeared to be ſome other more probable cauſe; thus, in one caſe where a vomit had been adminiſtered for the chincough, the patient had juſt before had the ſmall-pox; and in another inſtance, the patient had had a fall.— 13. *On the vapour baths of Ruſſia, conſidered as a means of preſerving health, and of curing ſeveral diſeaſes. By* Antonio Ribeiro Sanchéz, *formerly Firſt Phyſician to the Empreſs of Ruſſia.*—The chief object of this eſſay is to prove that the Ruſſian baths ſurpaſs, in utility and convenience, thoſe of the ancient Greeks and Romans, as well as thoſe of the modern Turks. The author firſt gives an hiſtorical account of the ſtructure and uſe of baths among the ancients, and compares them with thoſe of modern times. He obſerves, that the Turkiſh baths, ſuch as they are conſtructed in London, would be preferable to every other, if the air and the vapour of the room in which the patient ſweats, were frequently renewed, as in the Ruſſian baths. As he has ſeen baths of this kind at London, and alſo at Azoph, when that place was taken by the Ruſſians in 1736, he enters into a particular deſcription of them.

 His

His account of the Greek and Roman baths is collected chiefly from Vitruvius. The Russian bath, he obſerves, in which every thing is done in one apartment, is an epitome of the Roman and Turkiſh baths, which require four or five.

Of the Ruſſian baths, we are told, there are two ſorts, the one public, the other private; the latter differs from the former only in having a chamber, with beds in it, contiguous to the bath. Both are heated by means of a furnace in the middle of the bath. The upper part of the furnace is filled with pebbles, which are ſupported on an iron grate over the fuel, and kept conſtantly almoſt red hot. Around the ſides of the bath are ſeveral benches, riſing one above the other, like thoſe of an amphitheatre. When the wood in the furnace is reduced to aſhes, the funnel, through which the ſmoke paſſes, is cloſed, and the bath is then heated to a degree which is intolerable to perſons who have not been accuſtomed to it from their infancy. The bathers lie down naked on a ſtraw mattreſs, on the firſt or ſecond bench next the furnace; and by pouring cold water on the heated ſtones, the bath is inſtantly filled with a denſe hot vapour. This vapour, which may may be augmented or renewed at pleafure, foon brings on profufe perfpiration; and when the bather has fweated fufficiently, he is rubbed with foap and the branches of the lime tree, the leaves of which are covered with a down. He is next wafhed with warm water, and laftly with cold water; feveral pailfuls of which are poured on his head. They who bathe in the public baths, inftead of being wafhed in the bath before they quit it, plunge into fome pond or brook, in the open air, or roll themfelves in the fnow. They who make ufe of a private bath, have feveral pailfuls of cold water poured on their heads, and upon leaving the bath, go to bed in the room adjoining to it, which is moderately heated, and continue there till they have done fweating.

In the Turkifh and Roman baths, our author obferves, the vapour firft raifed remains unchanged, as in thofe there is no communication with the external air; while, in the Ruffian baths, the vapour is renewed every five or fix minutes. In thefe laft, the moment the perfon who bathes thinks he does not fweat fufficiently, or wants frefh air, he orders more water to be poured on the heated ftones, and the vapour inftantly paffes paſſes out through two windows, which are opened occaſionally for this purpoſe.

After deſcribing the manner of bathing, our author points out the abuſes committed by the Ruſſians in this reſpect. The firſt of theſe, we are told, conſiſts in their entering the bath too ſoon, that is, when it is filled only with the hot and dry ſmoke ariſing from the aſhes. This abuſe, he obſerves, is practiſed chiefly in the public baths, and by the lower ſort of people; and as it almoſt always occaſions more or leſs of head-ach, and thirſt, to allay which they often drink freely of cold water, its effects cannot fail to be injurious to health, and ſometimes are even ſuddenly fatal. As proofs of this, the author mentions two inſtances of perſons at Moſcow, who died in the bath, after drinking ice water while they were ſweating. Some, he obſerves, are ſo imprudent as to bathe immediately after a full meal. This he has found to be particularly hurtful to women, and in theſe he conſiders it as a frequent cauſe of irregular menſtruation, fluor albus, and ſterility. Some accuſtom themſelves to be cupped in the bath every month, or ſix weeks: this practice, he obſerves, is often pernicious. The habit of plunging into cold water, or ſnow, he thinks, thinks, can be allowable only in very robuſt habits; and he condemns the cuſtom of expoſing children, almoſt as ſoon as they are born, to the ſuffocating heat of a bath, filled with ſmoke; for it ſeems, that in Ruſſia, as ſoon as a lying-in woman is able to walk, which is generally within twenty hours after delivery, ſhe is obliged, by eſtabliſhed cuſtom, however inclement the ſeaſon may happen to be, to go to a bath, where ſhe and her infant undergo the uſual proceſs of ſweating, rubbing, and cold bathing.

Of the efficacy of theſe baths, when properly adminiſtered, in different diſeaſes, the author ſeems to entertain a very high opinion, particularly in the lues venerea, a diſeaſe to which, he thinks, three-fourths of the chronic complaints that prevail in Ruſſia owe their origin. —14. *On the miliary fever which prevails frequently in different parts of Normandy. By M.* Varnier.—15. *Account of an aneuriſm of the axillary artery. By M.* de Horne.—The ſubject of this caſe was a ſtout, active man, fifty years old, and extremely irraſcible. About ten years before his death, he was unuſually agitated by a violent fit of paſſion; and a few days after complained of a pulſation of the right ſubclavian vian artery. This ſeems to have been the beginning of the aneuriſm; which went on ſlowly increaſing, and, at the end of about eight years, appeared externally forming a tumour of about the ſize of a walnut. In the courſe of the two ſucceeding years, it acquired a prodigious bulk, ſo as to occupy the whole of that part of the thorax which is covered by the great pectoral muſcle, and extending likewiſe under the axilla to the lower coſta of the ſcapula. At length, in March 1774, two years after the appearance of the aneuriſmal tumour, the patient died ſuddenly, and the tumour inſtantly diſappeared. The dead body was opened by M. Sabatier. The aneuriſm was found to be of the ſubclavian artery, at that part where it paſſes out of the thorax, and begins to be diſtinguiſhed by the name of axillary artery. The ſecond and third of the true ribs, on which the preſſure of the aneuriſm had been moſt conſiderable, were become ſo extremely thin, that they had given way, and were found broken. It was to this fracture that the ſudden and fatal extravaſation of blood into the cavity of the thorax, which occaſioned the patient's death, was owing; the ends of the fractured bones having lacerated the ſac. M. de Horne

ſuppoſes

ſuppoſes the effect of the tumour on the ribs to have been purely mechanical, as no appearance of caries could be diſcovered. He has thought it right to obſerve, that at the time of the fit of paſſion, to which the origin of the diſeaſe was aſcribed, the patient was under a courſe of mercury. — 16. *Inquiries and obſervations concerning the Eſſential Epilepſy, or* morbus ſacer *of Hippocrates. By M.* Saillant. — This writer gives the name of *eſſential epilepſy* to that ſpecies of the diſeaſe, the ſeeds of which he ſuppoſes the patient to bring into the world with him. This original prediſpoſition, he obſerves, may be hereditary, although it may have remained dormant during one or more generations; or it may be acquired *in utero* from the paſſions of the mind, ſtate of the fluids, &c. of the mother during pregnancy; and he quotes examples from authors in proof of its having been tranſmitted by the mother, in conſequence of frights, &c. although ſhe herſelf has not been affected with the diſeaſe. We need hardly point out how repugnant theſe quotations are to the preſent ſtate of our knowledge with regard to the fœtus in utero. M. Saillant allows, that the diſeaſe may be owing to original mal-conformation of the ſolids, but he thinks it is more

 commonly commonly the effect of a depraved ſtate of the fluids; and his remarks, therefore, are confined chiefly to the diſeaſe, as ariſing from this cauſe.—17. *On the inconveniences of Stables, when badly conſtructed. By Abbé* Teſſier.——18. *Hiſtorical account of the Epizootic diſeaſe, which prevailed in Picardy in* 1779. *By M.* Vicq D'Azyr. — 19. *On the Glanders. By M.* Chabert, *Director and Inſpector General of the Royal Veterinary School.*—In this paper, the author endeavours to prove, that the glanders is a contagious diſorder; that it is ſporadic, and rarely epidemic. The horſe, mule, aſs, and zebra, are the only animals in which he has obſerved it.—20. *On the irritability of the Lungs. By M.* Varnier.—21. *Reflections on the intention of Nature in the ſtructure of the bones of the Cranium, peculiar to new-born Children; being an Eſſay on the advantages which may be attributed to that conformation. By M.* Thouret.—This writer ſuppoſes that the ſutures not only ſerve to leſſen the bulk of the child's head, in its paſſage through the pelvis, but that by the equal compreſſion of the brain, which is the conſequence of this diminution of bulk, they are the means alſo of rendering the child torpid and inſenſible. — 22. *Obſervations on the phenomena*

*which*

*which the urine exhibits in a ſtate of health. By M.* Hallé.—Phyſiologiſts have diſtinguiſhed the urine diſcharged immediately after drinking, from that which is voided ſeveral hours after a meal. The former, which is a watery ſaline excretion, that depoſits no ſediment, they have termed *urina potûs*; the latter they have ſtiled *urina ſanguinis*; and it is this to which M. Hallé has chiefly confined his reſearches. The reſults of his obſervations are, that the ſubſtances ſeparable from urine by a ſpontaneous decompoſition, are, 1. a gelatinous ſubſtance, or mucus, which forms the firſt ſediment; 2. a ſaline earthy ſubſtance; 3. a colouring ſubſtance; and 4. ſalts of different kinds.—23. *Of the analyſis and properties of the various conſtituent parts of Ipecacuanha. By Meſſieurs De Laſſone,* junior, & *Cornette.*—From theſe inquiries we learn, that the woody part of the root of Ipecacuanha is nearly as emetic as that which is ſeparated from it; and that an extract, prepared by infuſing the powder of this part in cold diſtilled water, poſſeſſes the ſame property, though in a milder degree. Theſe writers obſerve alſo, that the reſin and extract prove emetic in the ſame doſes, viz. of five or ſix grains; but that the former is generally more purgative than the the latter.—24. *Chemical inquiries concerning the processes hitherto employed in the preparation of Emetic Tartar. By M.* Caille.—25. *Observations and researches on the use of the Loadstone in Physic; being an Essay on Medical Magnetism. By Messieurs* Andry *and* Thouret.—The first fifty pages of this paper relate to the history of medical magnetism. From this account we learn, that the first Greek writer who mentions the topical use of the loadstone, is Aetius, who speaks of its efficacy in gout and convulsions. The Greeks are supposed to have borrowed this remedy from the Egyptians. Neither Celsus, Pliny, or Galen, have written any thing concerning it. Alexander Trallianus asserts, that it removes pain, when worn about the neck; and Hali Abbas, according to Zwinger, attributes similar properties to it; as does Marcellus, who practised at Bourdeaux about the year 338.

Paracelsus, we are told, who often had recourse to the loadstone in nervous affections, entertained an opinion that it acts on the fluids and viscera; and Father Kircher relates, that, in his time, it was the custom in Holland, in cases of hernia, to make the patient swallow filings of iron, and then to apply to the hernia a plaister, a plaifter, compofed of powdered loadftone, for eight days; during which time the patient remained in a proper pofture. This procefs was founded on a fuppofition, that the efforts of the iron and magnet to unite, would be favourable to the reduction of the hernia. About the beginning of the laft century, when a tafte for experimental philofophy began to difplay itfelf, the medicinal properties of the magnet were not overlooked. Bonetus mentions a cafe of furor uterinus, in which a cure was effected by a loadftone worn at the pit of the ftomach. In the German Ephemerides for 1686, we have an account of its utility in a cafe of gutta ferena; and in the *Mercure de France* for 1726, is related the cafe of a Monk, who, by wearing a loadftone, experienced fudden relief in a fpafmodic complaint, to which he had been fubject for feveral years.

The difcovery of the means of making artificial magnets, and of giving them a power fuperior to natural ones, gave rife to more inquiries on the fubject. In 1765, Dr. Klarich, of Gottingen, afcertained its efficacy in toothach, and alfo tried it in pains of the limbs, deafnefs, and palfy. In 1767, Dr. Weber communicated to the Royal Society of Gottingen, an an account of a painful affection of the eye, in a man aged 72, which had been cured in fixteen days, by applying a magnet three times a day, for the fpace of an hour each time, to the corner of the eye. In the fame year, he publifhed this and three other cafes at Hanover, in a work, entitled, *Die wurgkung des kunftlichen magnets*, &c. The firft of the three additional cafes related in this work was a cafe of ophthalmia; the fecond was a cafe of gutta ferena, and head-ach; and the third was a great weaknefs of the right eye. In all of thefe inftances a cure was obtained. In 1772, Profeffor Ludwig printed a Thefis at Leipfic, entitled, *De magnetifmo in corpore humano*, in which feveral facts relative to its efficacy in the cure of difeafes are ftated. In 1774, it obtained additional reputation, through the means of Father Hell, of Vienna, who, having applied himfelf to this fubject, made fome excellent artificial magnets, with which he fucceeded in a variety of cafes, chiefly of painful and fpafmodic affections. In thefe inftances, the magnets were worn conftantly by the patients for a confiderable length of time, at the pit of the ftomach, at the wrift, by way of bracelets, or in other forms. Father Hell, we are told, preferred a round fhape to any other. In France, it feems, the the Abbé le Noble has particularly diſtinguiſhed himſelf by the perfection to which he has brought the artificial magnet, and by his application of it to medical purpoſes. One of thoſe which he has made, although it weighs only fifteen pounds, ſupports a weight of two hundred and thirty. In 1777, the Abbé requeſted the Society to try the effects of his magnets in diſeaſes; and the authors of the paper now before us, which is the reſult of their enquiries, were appointed to make experiments on the ſubject.—Before they relate the caſes in which they have tried the loadſtone, they deſcribe the methods of applying it.—The moſt common, which is what the Abbé calls the application *en armure*, conſiſts in applying ſmall bars of artificial magnet, of about an inch long, four tenths of an inch broad, and one and a half tenth of an inch thick, to different parts of the body, in the ſhape of bracelets, garters, necklace, bandage round the head or waiſt, &c. The bracelets are formed of five of theſe bars; the garters of twelve, and the necklaces of ten. They are all of them covered with black velvet, and ſecured by means of ribbands. Inſtead of theſe bars, magnetic plates *(des plaques aimanteés)* are

ſometimes

ſometimes applied naked to the ſkin, and their ſhape is adapted to the part of the body on which they are placed.

The firſt caſe in which our authors tried the effects of magnetiſm, has been in part related in the firſt volume of the Memoirs of the Society (*See our 3d vol. p.* 158). In that caſe, we are told, the pain has continued to return at times, with more or leſs violence, but is conſtantly alleviated by the application of a loadſtone. Several other inſtances are added of its effects in tooth-ach, rheumatiſm, cramps, palpitations, and epilepſy; but in all of theſe complaints its effects ſeem to have been only palliative. The reſults of the inquiry, however, as given by the authors, are, 1. that the loadſtone poſſeſſes a ſalutary property; 2. that this property is independent of its coldneſs, weight, or metallic properties; 3. that it ſeems to act on the nerves, but has no apparent influence on the fibres, fluids, or viſcera; 4. that it may be ranked among the antiſpaſmodics.——26. *Obſervations on the properties of the Bark of a Tree, known at Madagaſcar by the name of* Bela-Aye. *By M.* Sonnerat.—We have already had occaſion to mention this bark (*ſee vol.* III. *p.* 421). Our author, who brought brought a confiderable quantity of it from Madagafcar, was not able to procure either the flower or the fruit of the tree that produces it. Adminiftered twice a day, in dofes of twenty-four grains, it is faid to have cured a chronic diarrhœa, which had refifted a variety of remedies for the fpace of fix years.

